# Alternate partial root zone subsurface drip irrigation increases alfalfa water production function by increasing hay yield, rather than actual crop evapotranspiration

**DOI:** 10.3389/fpls.2026.1909849

**Published:** 2026-08-26

**Authors:** Yadong Wang, Yansong Li, Qiuchi Zhang, Kai Gao, Derong Su

**Affiliations:** 1 College of Grassland Science, Inner Mongolia Minzu University, Tongliao, China; 2 College of Grassland Science, Beijing Forestry University, Beijing, China

**Keywords:** crop water production function, partial root zone drying, perennial forage, regulated deficit irrigation, water consumption

## Abstract

Both conventional subsurface drip irrigation (CI) and alternate partial root zone subsurface drip irrigation (AI) are water-saving irrigation methods (IMs) used to solve agricultural water crises. The responses of alfalfa (*Medicago sativa* L.) actual evapotranspiration (ETa), and the relationship with hay yield to the two types of IMs have not been evaluated, which is crucial for the sustainable agricultural development of irrigation and water management. We investigated the effects of CI and AI and three irrigation volumes (IVs) (10, 20, and 30 mm) on alfalfa ETa, and their relationship with hay yield in 2017 and 2018. AI significantly reduced the alfalfa ETa by 10 and 17% under 20 and 30 mm irrigation volumes, respectively, in 2017 compared with CI. However, AI increased the ETa by 42 mm under 10 mm irrigation volume in 2017 and by 43 mm under a 30 mm irrigation volume in 2018. There was no significant effect on the alfalfa ETa between CI and AI under 10 and 20 mm IVs in 2018. The changes in alfalfa ETa could be explained by soil moisture and soil water storage dynamics. In addition, alfalfa ETa is also affected by irrigation volume and stand year. Over more, hay yield was linearly correlated with the alfalfa ETa, and the increase in the slope of the alfalfa–water production function of AI compared to the equivalent slope of CI was 180%. The increase is largely explained by the reproduce in hay yield. This study provides a better alfalfa–water production function irrigation method for promoting AI technology applied in farm production.

## 1 Introduction

The world is facing a huge crisis in the use of fresh water, of which agriculture is the main user (FAO, 2025). It is estimated that agricultural irrigation needs will increase in the future (FAO, 2021). Therefore, water-saving irrigation technologies are being increasingly used to solve the problem of agricultural water shortage (Romero et al., 2012; Wang et al., 2021; Ward and Pulido-Velazquez, 2008; Xiao et al., 2015). Without reducing the irrigation volume, the popularization and application of water-saving irrigation technology will not only improve crop yield but also reduce crop transpiration and soil evaporation loss (Hong et al., 2021; Xie et al., 2020; Yang et al., 2020; Zhang et al., 2022). Actual crop evapotranspiration (ETa) refers to the total amount of soil moisture consumed by crops through evaporation and transpiration within a certain period of time (Cao et al., 2021; Hong et al., 2021; Schwartz et al., 2019). The relationship between ETa and yield is often used to characterize the crop–water production function (Çolak et al., 2018; Gençoğlan et al., 2006). Therefore, comparing the ETa, and the relationships with yield become increasingly important under the implementation of water-saving irrigation technology, which is crucial for the sustainable agricultural development of guiding crop irrigation and water management.

How to develop irrigation agriculture under limited water resources is still a dilemma (Chen et al., 2021; Ding et al., 2019; Li et al., 2021; Vogeler et al., 2016; Wang et al., 2018; Zhou et al., 2020). Therefore, many water-saving irrigation methods (IMs) in irrigated agriculture, such as furrow irrigation, subsurface drip irrigation, and partial root zone drying, have emerged (R. Liu et al., 2021; Romero et al., 2012; Wang et al., 2021; Xiao et al., 2015). Among them, subsurface drip irrigation is the most water-saving irrigation method (Fu et al., 2021; Gao et al., 2021; Hondebrink et al., 2017; Wang et al., 2021). However, conventional subsurface drip irrigation (CI) also has its disadvantages; for example, the distance between drip pipes is too far in the conventional irrigation system, and a large irrigation volume is required to make the water migrate to all root zones (Alam et al., 2002; Appels and Karimi, 2021; Kandelous et al., 2012; Lamm et al., 2012; Rodríguez-Sinobas et al., 2021). The introduced alternate partial root zone subsurface drip irrigation (AI) technology can effectively resolve this problem by alternating on both sides of the root system (Wang et al., 2021, Wang et al., 2024, Wang et al., 2025). This has the disadvantage of increasing ETa and reducing yield for the application of AI technology compared with CI (Çolak et al., 2018). However, another study showed that AI did not change the advantages of ETa compared to CI (Sun et al., 2024). Another study has shown that the ETa and yield of one-year-old alfalfa are reduced under AI, while the ETa of two-year-old alfalfa significantly increases under AI compared with CI, and the yield also increases but without significant differences between the treatments (Wang et al., 2021). The three field comparative studies have not reached a relatively consistent conclusion (Çolak et al., 2018; Wang et al., 2021, Wang et al., 2024). Wang et al. (2021) only compared the alfalfa ETa and hay yield under full irrigation. Although they later compared the yield differences between different AI and CI (Wang et al., 2024), we still have a great deal of uncertainty regarding the comparison of ETa under different irrigation methods and irrigation volume interactions. And to explain the main influencing factors of these changes, namely, changes in soil water storage dynamics and changes in soil moisture.

Alfalfa (*Medicago Sativa* L.), a common forage species, is the most widely planted perennial leguminous forage worldwide. However, due to the large ETa of alfalfa compared with other crops (Bacenetti et al., 2018; Cui et al., 2018; Jiang et al., 2015), it becomes more important to study the relationship between ETa and yield. The crop–water production function is an expression that describes the mathematical relationship between crop yield and ETa (Çolak et al., 2018; Jiang et al., 2012; Wang et al., 2020). Previous studies have found that the crop–water production function is usually linear (D. Zhang et al., 2021, Zhang et al., 2013) or quadratic (Jiang et al., 2012; Wang et al., 2020). This uncertainty also appears in alfalfa research (Li et al., 2023; Lindenmayer et al., 2011; Montazar and Sadeghi, 2008; Rogers et al., 2016; Wang et al., 2021). This uncertain alfalfa–water production function is not conducive to field management. The irrigation method is an important field management factor affecting the crop–water production function (Çolak et al., 2018; Gençoğlan et al., 2006). Previous studies have shown that the effects of CI and AI have different responses in green bean (Gençoğlan et al., 2006) and eggplant (Çolak et al., 2018). Studies on green bean have shown that AI increases the slope between yield and ETa compared with CI (Gençoğlan et al., 2006). This effect shows opposite results in eggplant (Çolak et al., 2018). The inconsistency between the results of the two field studies shows the uncertainty of the relationship between yield and ETa under different irrigation methods. In addition, the alfalfa–water production function relationship between CI and AI in water management has yet to be verified.

The Hexi Corridor is an arid area located in the northwest of China with limited water resources, scarce precipitation, and a large ETa, and it is a major alfalfa production area in China (Wang et al., 2020; Wang et al., 2021; Zhou et al., 2020). Here, a two-year field experiment was conducted to investigate the effects of AI and CI on alfalfa ETa, and the relationship with hay yield under different irrigation volumes in this area. In addition, we studied the soil moisture and soil water storage dynamics to explain the changes in alfalfa ETa. We focused on two issues: (i) the effects of two IMs on alfalfa ETa and hay yield under three irrigation volumes, use the dynamic changes in soil moisture and soil water storage during the process to explain the differences in ETa among different treatments; and (ii) the effects of different IMs on the alfalfa–water production function, explain whether these changes are primarily driven by hay yield or ETa. This research is significant in providing insights into the effects of different IMs on alfalfa production in an arid region with limited water resources.

## 2 Materials and methods

### 2.1 Site description and experimental design

The experiments were carried out during the growing seasons of 2017 and 2018 at the National Field Scientific Observation and Research Station of Oasis Agroecosystem (35°52′ N and 102°50′ E, altitude 1581 m), located in Wuwei in Gansu Province, China. The soil of the field was sandy loam, with an average field capacity of 0.29 cm^3^ cm^−3^, a wilting point of 0.09 cm^3^ cm^−3^, and a soil bulk density of 1.50 g cm^−3^ in the upper 1.6 m of soil.

**Figure 1:**
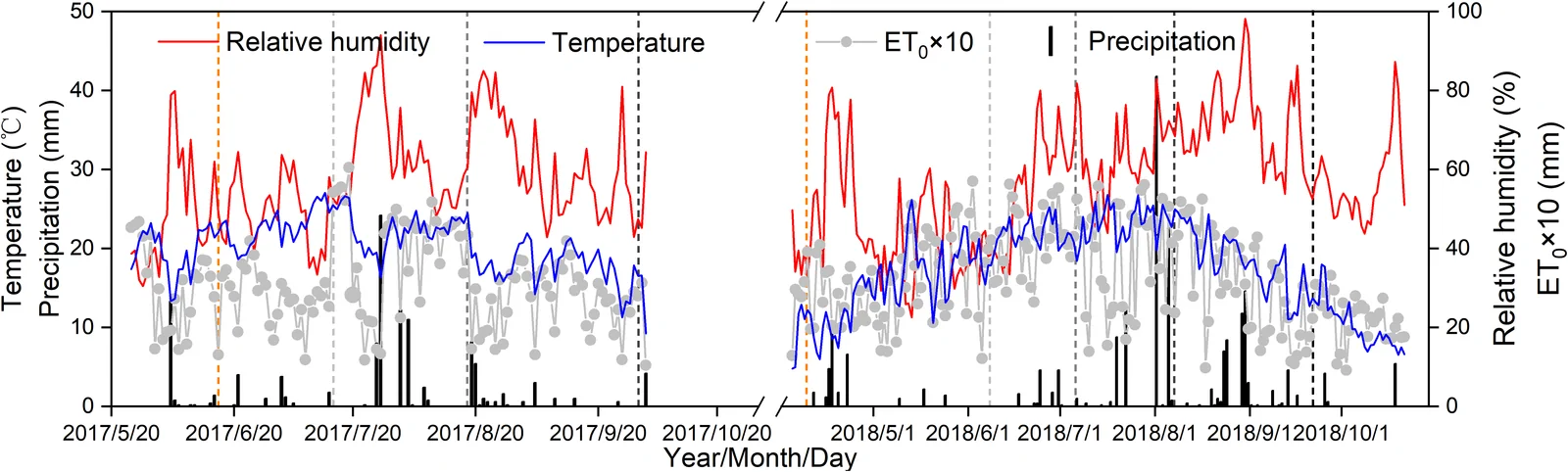
Meteorological data and reference crop evapotranspiration (ET_0_) (Allen et al., 1988) in the experimental years. The dotted vertical orange line indicates the beginning of irrigation, and the dotted vertical gray line indicates the four harvests. For interpretation of the references to color in this figure legend, the reader is referred to the web version of this article.

The mean annual precipitation and pan evaporation in this region are 164 mm and 2000 mm, respectively. The mean annual temperature is 8.8 °C, and the annual accumulated temperature (>0 °C) is 3550 °C. The average temperature, daily precipitation, and daily relative humidity (%) during the 2017 and 2018 alfalfa growing seasons are shown in **Figure 1**. The precipitation received during the growing seasons was 109 and 192 mm in 2017 and 2018, respectively (**Figure 1**). In addition, the daily reference crop evapotranspiration (ET_0_) (Allen et al., 1988) for 2017 and 2018 is shown in **Figure 1**.

In the experiments, AI (n = 3) and CI (n = 3) were compared under three different irrigation volumes (IVs) (**Table 1**), and 18 plots were set up. The size of the plot was 6 m long and 4 m wide. The frequency of subsurface drip irrigation was once a week (Wang et al., 2021). Due to excessive precipitation, schedules for July 29 and August 19 in 2017 and for August 8, August 19, August 26, and September 2 in 2018 were all delayed by 1 week. Accordingly, there were 13 irrigation events in 2017 (except for seedling irrigation and winter irrigation) and 19 irrigation events in 2018 (**Table 1**). The total annual IVs were 180, 310, and 440 mm in 2017 and 190, 380, and 570 mm in 2018 for each treatment (**Table 1**).

**Table 1:** Alfalfa hay yield as affected by irrigation method (IM) (AI and CI), irrigation volume (IV) (10 mm, 20 mm, and 30 mm), and their interaction (IM×IV) at three harvests in 2017 and four harvests in 2018.

Treatment	2017			2018				2017	2018
	1st	2nd	3rd	1st	2nd	3rd	4th		
CI+10 mm	3050.40d	2946.67cd	2072.93e	1781.33b	1320.67c	837.07c	1530.40d	8070.00e	5469.47d
CI+20 mm	3391.73cd	3270.40bc	3109.47c	2508.53b	1497.07c	1498.67b	1861.20cd	9771.60c	7365.47c
CI+30 mm	5825.47a	3995.60a	3935.80a	3886.67a	3038.93a	2752.13a	3065.60a	13756.86a	12743.33a
Average	4089.20B	3404.22A	3039.40A	2725.51B	1952.22B	1695.96B	2152.40B	10532.82A	8526.09B
AI+10 mm	3694.40c	2594.63d	2695.83d	2234.53b	2212.67b	1574.53b	2156.53bc	8984.86d	8178.27c
AI+20 mm	4307.07b	3021.47bcd	3081.60c	3682.27a	3239.07a	2420.27a	2425.07b	10410.13c	11766.67b
AI+30 mm	5717.47a	3517.33ab	3509.20b	4415.33a	3212.00a	2490.40a	3457.87a	12744.00b	13575.60a
Average	4572.98A	3044.48B	3095.54A	3444.04A	2887.91A	2161.73A	2679.82A	10713.00A	11173.51A
Tukey-adjusted_0.05_									
IM	**<0.001**	**0.0003**	0.392	**0.002**	**<0.001**	**<0.001**	**<0.001**	0.149	**0.001**
IV	**<0.001**	**<0.001**	**<0.001**	**<0.001**	**<0.001**	**<0.001**	**<0.001**	**<0.001**	**<0.001**
IM×IV	**0.0006**	0.0632	**<0.001**	0.237	**<0.001**	**<0.001**	0.430	**<0.001**	**<0.001**

Different uppercase letters indicate significant differences between irrigation methods (*P* < 0.05). Different lowercase letters indicate significant differences among six treatments, according to Tukey-adjusted (*P* = 0.05). 10 mm, 20 mm, and 30 mm represent the three treatments under alternate partial root zone subsurface drip irrigation (AI), and conventional subsurface drip irrigation (CI). Bold values represent statistically significant differences.

**Figure 2:**
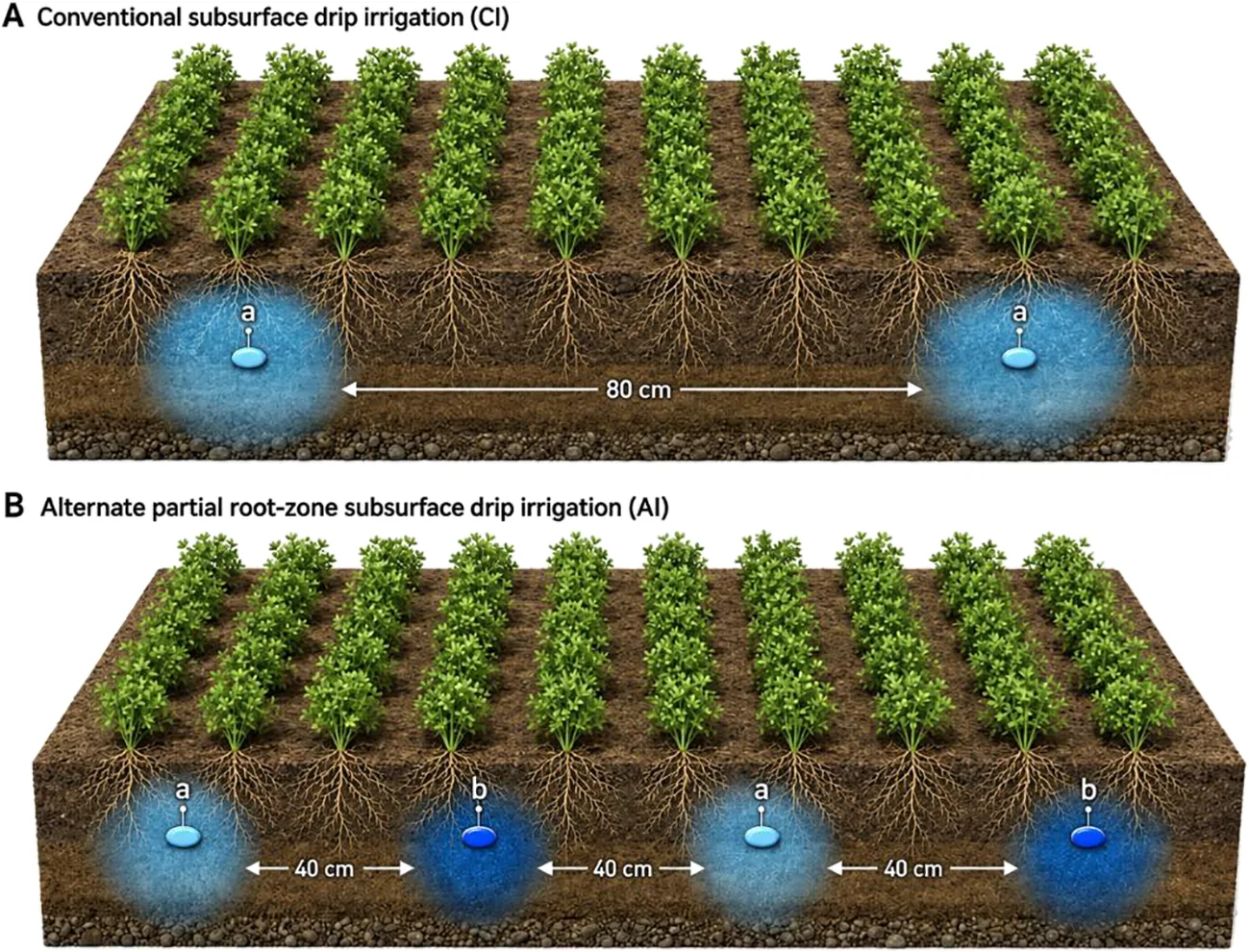
Layout of the irrigation system for CI **(A)** and AI **(B)**. a and b are SDI systems irrigated in turn. For interpretation of the references to color in this figure legend, the reader is referred to the web version of this article.

The subsurface drip irrigation systems were used to implement irrigation settings. The subsurface drip irrigation systems were buried at a depth of 0.2 m before the alfalfa was sown on the subplots. Inline emitters (DAYU Water-saving Group Co. Ltd., Gansu, China) with a discharge rate of 3 L h^−1^ were installed at 0.3 m intervals. Two groups of subsurface drip irrigation units were nested in a test subplot, and subsurface drip irrigation was realized using manual valves on distribution pipes (**Figure 2A**). The CI was established by one set of subsurface drip irrigation systems (**Figure 2A**). The AI systems were established by two sets of subsurface drip irrigation systems (**Figure 2B**), and detailed information was presented by Wang et al. (2021), who used two sets of subsurface drip irrigation systems in two irrigation events: a) subsurface drip irrigation system used for 1, 3, 5… irrigation events and b) subsurface drip irrigation system used for 2, 4, 6… irrigation events (**Figure 2B**).

Alfalfa (cv. MF4020) was planted at the standard of 20 kg hm^−2^ on May 20, 2017, by drilling, with a sowing depth of 0.05 m and a row space of 0.2 m (**Figure 2**). The seedlings were irrigated with micro-sprinklers, with the irrigation volume of 10 mm each time and twice a week. The irrigation experiment started on June 24, 2017 and May 8, 2018 and ended on September 30, 2017 and September 20, 2018, respectively. The plots were harvested three times (July 15, August 18, and September 30) during the growing season in 2017 and four times (June 7, July 5, August 7, and September 20) in 2018. Pest control was applied as per best management practices throughout the experiment, and weeds were removed manually after each harvest.

### 2.2 Measurements

Every 2–3 days, the Diviner 2000 system (Sentek Pty Ltd., Australia) was used to monitor the soil moisture in the vertical soil layer below 1.6 m (every 0.1 m) of the surface of each plot. Two Diviner 2000 system tubes were placed in each subplot has described in detail at the previous research (Wang et al., 2021). In addition, the gravimetric soil moisture was measured via the oven-drying method and used to calibrate the data for the Diviner 2000 system (Sentek Pty Ltd., Australia) at each harvest stage.

Alfalfa hay yield was determined by combining a large plot (1 m × 1 m) with a small plot (20 cm × 20 cm). The moisture content of alfalfa was measured in a small plot, and the hay yield of 1 m × 1 m sample plot was calculated based on fresh weight with a large sample plot. Five small plots were randomly selected from the test area, and the yield of fresh forage was determined immediately after harvest. Then, the samples from the small plot were placed in an oven for 1 h at 105 °C for sterilization. Alfalfa hay yield was determined after oven drying at 65 °C to a constant weight.

### 2.3 Calculation

The actual crop evapotranspiration of each harvest was estimated using the soil water balance method, which involved assessing the changes in the soil moisture in the 0–1.6 m soil layer over a period of time, as showed in Equation 1:

(1) ETa=I+P+S−ΔW−D

where ETa is the actual crop evapotranspiration (mm), *δ*W is the change in soil water storage (mm), P is the precipitation (mm) recorded by the HOBO automatic weather station, I is the irrigation water volume (mm), S is the supplement of ground water (mm), and D is deep percolation (mm). At the experimental site, the groundwater contribution was negligible because the groundwater table was deeper than 25 m (S = 0). Additionally, in this experiment, the subsurface drip irrigation system delivered a controlled irrigation volume (D = 0).

### 2.4 Statistics

Initial soil water storage, ultimate soil water storage, soil water storage change, ETa and hay yield were analyzed with one-way and two-way analysis of variance using IBM SPSS Statistics (IBM Corp., Armonk, NY, USA). All figures were drawn using Origin 2022 software (Origin Lab Corp., Northampton, MA, USA).

## 3 Results

### 3.1 Alfalfa actual crop evapotranspiration

**Figure 3:**
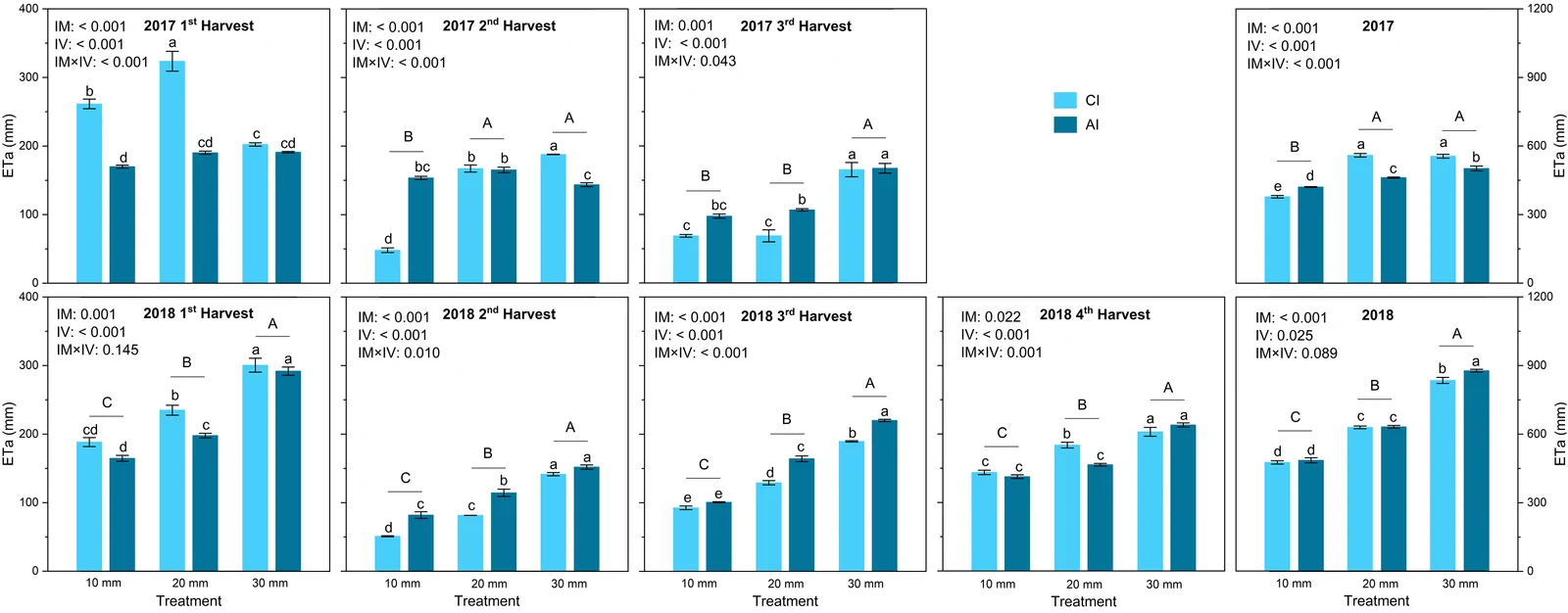
Alfalfa actual crop evapotranspiration (ETa) as affected by irrigation method (IM), irrigation volume (IV), and their interaction (IM*IV) at three harvests in 2017 and four harvests in 2018. 10 mm, 20 mm, and 30 mm represent the three treatments under alternate partial root zone subsurface drip irrigation (AI1, AI2, and AI3), and conventional subsurface drip irrigation (CI1, CI2, and CI3). Different uppercase letters indicate significant differences among irrigation volumes (*P* < 0.05). Different lowercase letters indicate significant differences among six treatments, according to Tukey-adjusted (*P* = 0.05). For interpretation of the references to color in this figure legend, the reader is referred to the web version of this article.

The IMs and IVs significantly affected the ETa of alfalfa in 2017–2018 (*P* < 0.05), and the interaction between IMs and IVs only affected the alfalfa ETa in 2017 but had no significant effect on the alfalfa ETa in 2018 (*P* = 0.089) (**Figure 3**). A linear mixed-effects model also showed that irrigation method had no significant effect on ETa (*P* = 0.872) (**Supplementary Table 1**). In 2017, AI decreased the ETa by 98 and 54 mm under IVs of 20 and 30 mm, respectively, compared with CI, while AI increased the ETa by 43 mm compared with CI under IVs of 10 mm in 2017 and 30 mm in 2018. There was no significant difference between the two IMs under IVs of 10 and 20 mm in 2018.

There were significant differences in alfalfa ETa under the two IM and three IV treatments at seven harvests in two years (*P* < 0.05) (**Figure 3**). The linear mixed-effects model also showed that harvest time and stand year had a significant impact on ETa, with *P* values of <0.001 and 0.002, respectively (**Supplementary Table 2**). Significant differences in the IM and IV interaction were also found in the two years, except at the 1^st^ harvest in 2018 (*P* < 0.05) (**Figure 3**). In 2017, the CI2 treatment had the highest alfalfa ETa at the 1^st^ harvest, which was 133–154 mm higher than that in the AI treatments (**Figure 3**). CI1 had the lowest alfalfa ETa at the 2^nd^ harvest, which was 2.98–3.91 times lower than that in the other treatments. CI3 had the highest alfalfa ETa at the 2^nd^ harvest. At the 3^rd^ harvest, especially under 10 and 20 mm irrigation volumes, the alfalfa ETa of AI treatments was significantly higher than that of CI by 29–38 mm (*P* < 0.05). In 2018, at the 1^st^ harvest, with the same irrigation volume, the alfalfa ETa was 8–37 mm higher under CI treatment than under AI treatment. The alfalfa ETa of AI under 10 and 20 mm IVs was significantly lower than that of CI at the 1^st^ harvest. With the same irrigation volume, the alfalfa ETa was 11–33 mm lower under CI treatment than under AI treatment in 2018 at the 2^nd^ harvest. At the 3^rd^ harvest, the alfalfa ETa was 8–38 mm lower under CI treatment than under AI treatments. At the 4^th^ harvest, the alfalfa ETa was higher under CI than under AI with a 20 mm irrigation volume.

### 3.2 Alfalfa soil moisture and soil water storage

**Figure 4:**
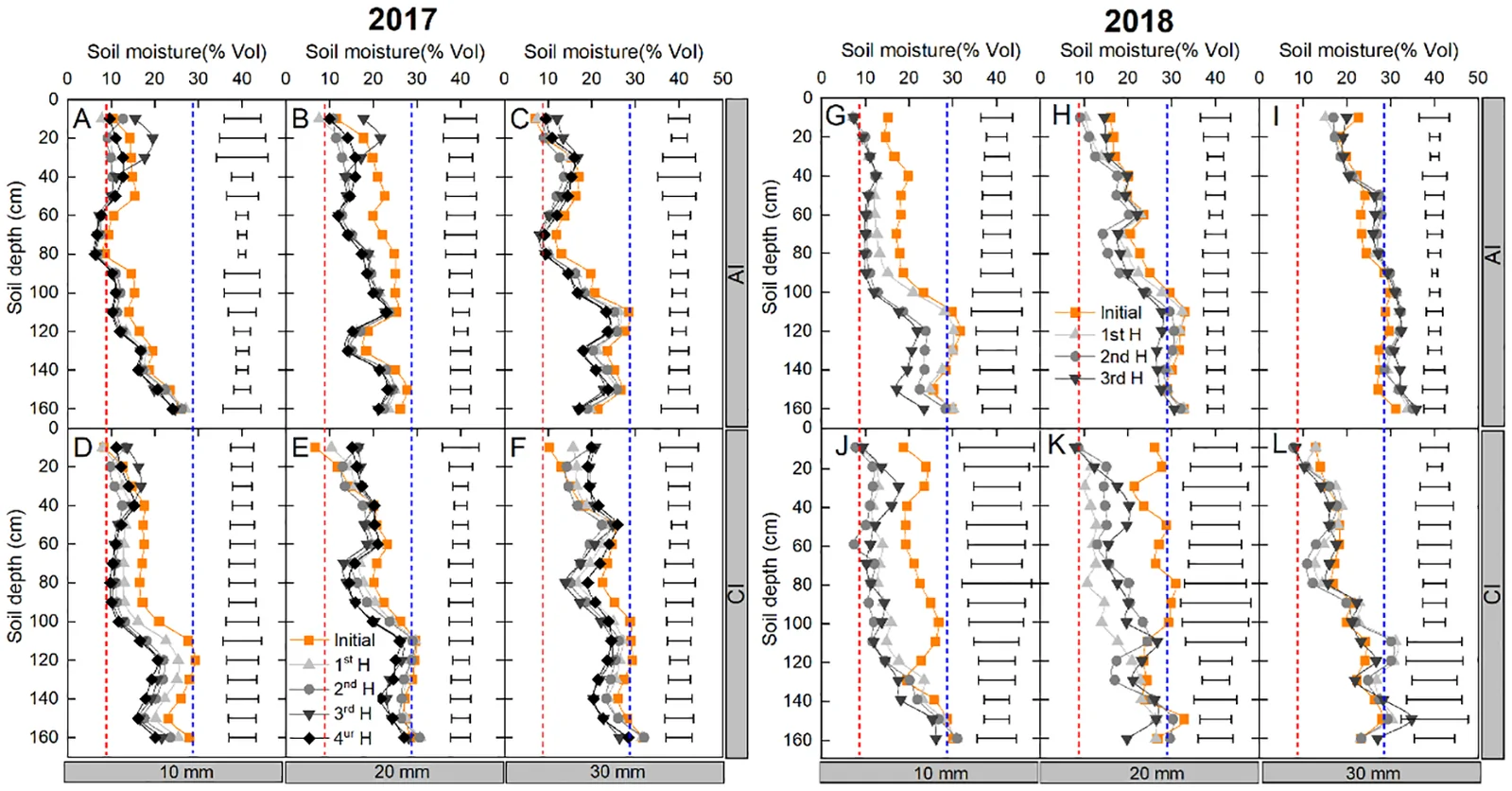
Alfalfa soil moisture at 10–160 cm soil depth at the initial and three harvests in 2017 and 2018. AI represents alternate partial root zone subsurface drip irrigation; CI represents convention irrigation with subsurface drip irrigation; and 10, 20, and 30 mm represent irrigation volumes at each irrigation event, respectively. The red dotted line indicates the average field water capacity, and the blue dotted line indicates the average wilting coefficient. The error bars denote the results of Tukey-adjusted (*P* = 0.05) of the significant differences among the initial and three harvests. For interpretation of the references to color in this figure legend, the reader is referred to the web version of this article.

The soil moisture at the depth of 10–160 cm with different IVs was compared at each harvest in 2017 and 2018 (**Figure 4**). Shallow soil layers (10–40 cm) and deep soil layers (50–160 cm) showed different trends. Each harvest soil moisture value in 2017 was higher than the initial value under the AI treatment with 30 mm irrigation volume, especially in the deep soil layers (**Figure 4**). Moreover, the compensation effect of a 30 mm irrigation volume in CI also occurred in 100–150 cm soil layers, mainly in the 1^st^ and 2^nd^ harvests (**Figure 4F**). The AI treatment soil water storage was 21 mm higher than the CI treatment soil water storage at the 3^rd^ harvest with a 20 mm irrigation volume (**Figure 4**). The AI treatment mainly used shallow soil layer moisture during the 1^st^ harvest and deep soil layer moisture at the 2^nd^ or even the 3^rd^ harvest with a 10 mm irrigation volume (**Figure 4A**). However, the soil moisture at depths of 10–120 cm and 10–100 cm decreased significantly in the CI treatments with IVs of 10 and 20 mm, respectively, at the 3^rd^ harvest (**Figures 4D, E**). In 2018, soil moisture decreased to various degrees, especially in the deep soil layers in all treatments (**Figure 4**). The deep soil moisture showed a decreasing trend with the increase in harvest regime, except for the CI treatment with a 30 mm irrigation volume. Compared with the 3^rd^ harvest, the soil moisture of the middle layers (50–100 cm) was compensated by the CI treatment with the 30 mm irrigation volume at the 4^th^ harvest, and the compensation volume was 19 mm (**Figure 4L**). In addition, the compensation effect of soil moisture was observed in shallow soil layers under the CI treatment with 20 and 30 mm IVs (**Figures 4K, L**). The soil water storage compensation amount was 7–24 mm among the four harvests (**Figures 4H, I**). Each irrigation treatment of AI showed water consumption in deep soil layers, and a water compensation effect occurred at the 3^rd^ harvest in shallow soil layers (**Figures 3G–I**). Compared with the initial water storage, AI increased by 0–10 mm in shallow soil layers (**Figures 4G–I**). By comparing the dynamics of shallow soil water storage under the two IMs with different irrigation volumes, it was found that the consumption of water in shallow soil layers under AI treatment was greater than that under CI treatment (**Figure 4**).

**Figure 5:**
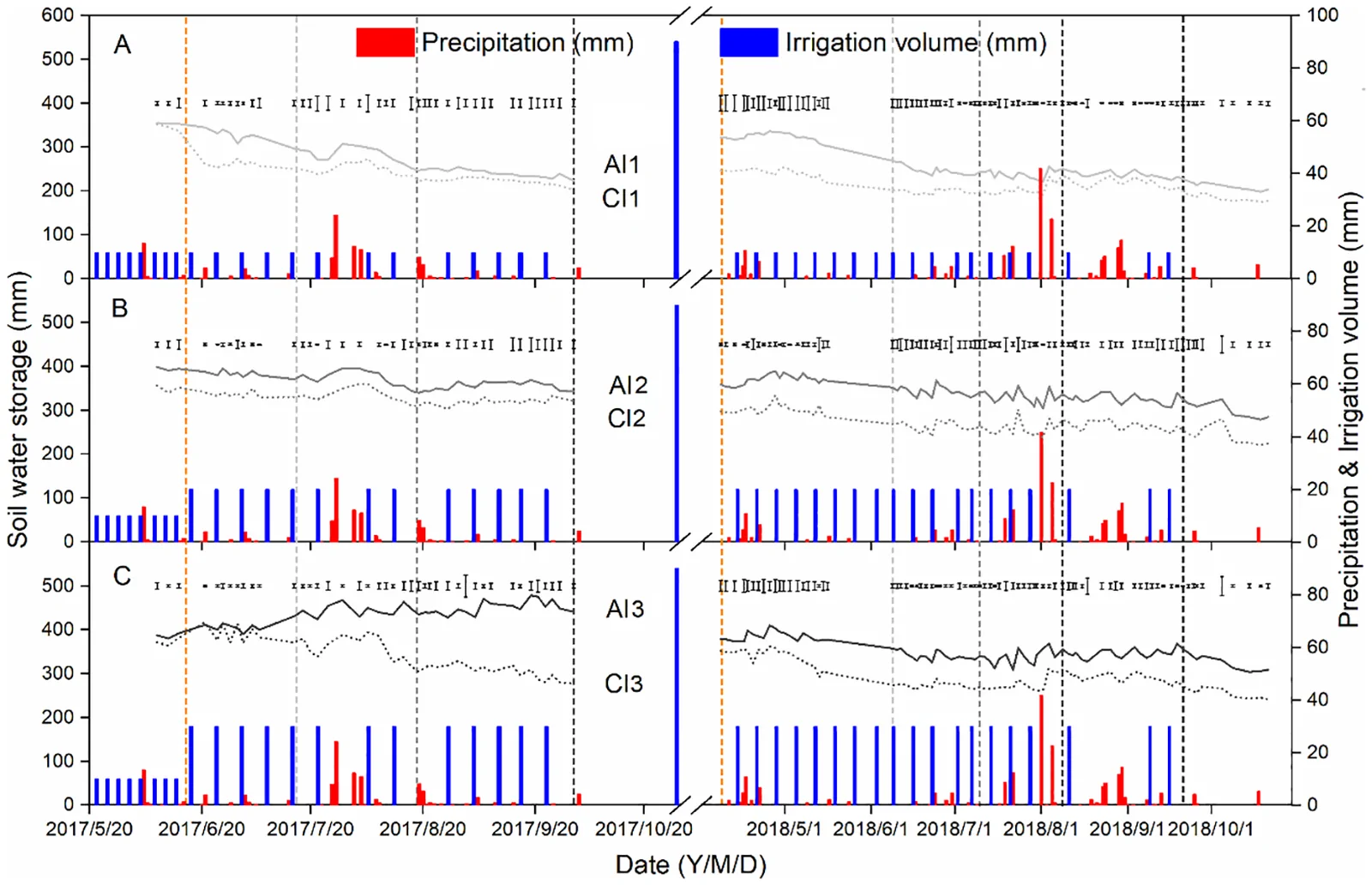
Soil water storage dynamics of AI (gray solid line) and CI (gray dashed line), precipitation (red bars), and irrigation volume (blue bars) at 10–160 cm soil depth during the growth period in 2017 and 2018. **(A)** represent the 10 mm irrigation volume under alternate partial root zone subsurface drip irrigation (AI) and conventional subsurface drip irrigation (CI). **(B)** represent the 20 mm irrigation volume under alternate partial root zone subsurface drip irrigation (AI) and conventional subsurface drip irrigation (CI). **(C)** represent the 30 mm irrigation volume under alternate partial root zone subsurface drip irrigation (AI) and conventional subsurface drip irrigation (CI). Winter irrigation occurred on October 29, 2017, and the irrigation volume was 90 mm. The vertical orange dotted lines indicate the initial stage, and the vertical dotted lines from shallow to deep indicate each harvest. The error bars denote the results of a t-test (P = 0.05) of the significant differences between AI and CI treatments. For interpretation of the references to color in this figure legend, the reader is referred to the web version of this article.

Two years of different IM and IV treatments showed that, except for the AI treatment with the 30 mm irrigation volume in 2017, soil water storage increased by 54 mm compared with the initial soil water storage (**Figure 5**). In the other treatments, the soil water storage decreased to varying degrees within two years (**Figure 5**). The reduction in soil water storage of the other treatments in 2017 was between 36 and 150 mm, and the reduction in 2018 was between 68 and 119 mm (**Figure 5**). In 2017, the treatment with the largest reduction in soil water storage of alfalfa in the whole year was CI1, while in 2018, it was AI1 (**Figure 5**). The effects of IM, IV, and their interaction on initial soil water storage, ultimate soil water storage, and changes in soil water storage at each harvest in 2017 and 2018 are shown in **Figure 5**. The impact of the IM on the initial soil water storage at the 1^st^ harvest in 2017 (*P* = 0.754) was not significant, and the interaction between IM and IV had no significant impact on the change in soil water storage at the 1^st^ harvest in 2017 (*P* = 0.154). Except for these, the IM, IV, and their interaction significantly affected the initial soil water storage, ultimate soil water storage, and soil water storage change (*P* < 0.05).

### 3.3 Alfalfa hay yield

IMs significantly affected the alfalfa hay yield in 2018 (*P* = 0.001) but not in 2017 (*P* = 0.149) (**Table 1**). However, the IVs and the interaction of IMs and IVs significantly affected the alfalfa hay yield in 2017–2018 (**Table 1**). A linear mixed-effects model also showed that IM had no significant effect on alfalfa hay yield (*P* = 0.991) but IV significantly affects alfalfa hay yield (*P* < 0.001) (**Supplementary Table 3**). Difference to ETa, hay yield was not influenced by the covariance between stand year and harvest time (**Supplementary Table 4**). In 2017, under the high irrigation volume of 30 mm, the improvement of AI on yield was slightly lower than that of CI. In the same year and in 2018 under other irrigation volumes, AI increased alfalfa yield by 6–60% compared with CI.

The reason for the insignificant effect of the above IM on alfalfa hay yield in 2017 is that the response of hay yield to IM at the 2^nd^ harvest showed the opposite of the consistency observed at other harvests (**Table 1**). The alfalfa hay yield of the AI under low IVs (10 and 20 mm) was slightly lower than that of the CI, although it did not reach a significant level (**Table 1**). There was no significant difference in the first crop of 2017 with a high irrigation amount of 30 mm; the alfalfa hay yield of the AI treatment was significantly lower than that of CI at the 2^nd^ and 3^rd^ harvests (**Table 1**). In 2018, AI showed a consistent increase in alfalfa hay yield by 25–116%. AI increased alfalfa hay yield by 14, 6, and 13% at the 1^st^, 2^nd^, and 4^th^ harvests, respectively, but decreased alfalfa yield by 10% at the 3^rd^ harvest compared with CI under a 30 mm irrigation volume.

### 3.4 Alfalfa–water production function

**Figure 6:**
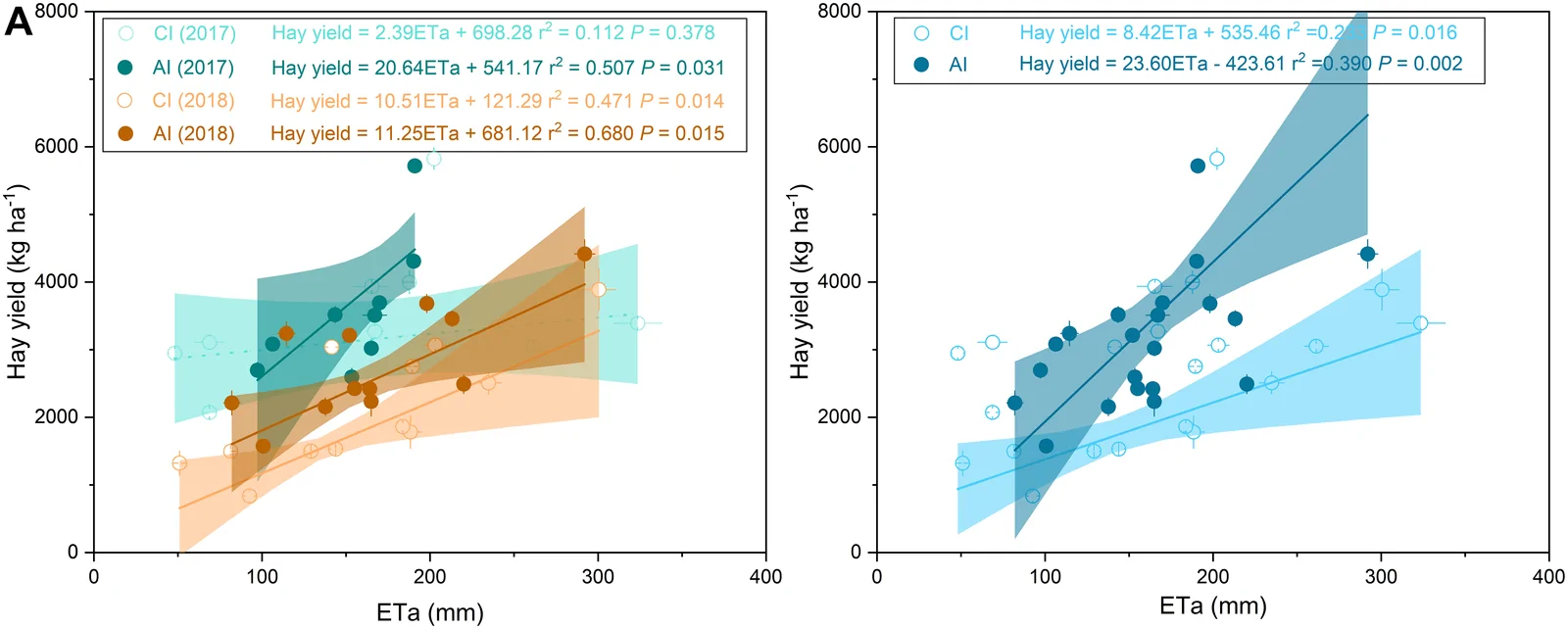
**(A)** shows the alfalfa–water production function at seven harvests under the CI and AI in 2017 and 2018, and **(B)** shows the alfalfa–water production function at seven harvests under CI and AI.

Significant linear relationships were established between the alfalfa ETa and hay yield for the CI and AI irrigation treatments (*P* < 0.01) (**Figure 6**). As shown in **Figure 6b**, the slope of the alfalfa–water production function was steeper for AI than for CI (23.60 *vs.* 8.42). Specifically, in 2017, the slope of the alfalfa–water production function under AI was 20.64, and it was 2.39 under CI. In 2018, the slope of the alfalfa–water production function was 11.25 under AI and 10.51 under CI (**Figure 6a**). Furthermore, analysis of covariance revealed that both IM and IV affect the water production function, but their interaction response has no significant effect (**Supplementary Tables 5**, **6**). The ETa and hay yield at the 95% confidence intervals under AI and CI were ±21.06, ± 15.90 and ±542.90, ± 162.03, respectively (**Supplementary Table 7**). Compared to CI, AI increased alfalfa hay yield, thereby increasing the water production function (**Figure 6b**; **Supplementary Table 7**).

## 4 Discussion

In 2017–2018, we evaluated the effects of two types of IMs (AI and CI) on ETa, and the relationship with hay yield under three IVs (10, 20, and 30 mm/event) at Hexi Corridor in northwest China. The changes in hay yield and alfalfa ETa were explained by soil moisture and soil water storage dynamics. Additionally, the slope between hay yield and alfalfa ETa was stronger for AI than for CI, indicating that AI may be a more efficient method for increasing the alfalfa–water production function for water management.

### 4.1 Alfalfa ETa under CI and AI

Comparing the alfalfa ETa in the two IMs (CI and AI) showed that the one-year-old alfalfa ETa could be increased by 54–98 mm under IVs of 20 and 30 mm in AI (**Figure 3**). The change in soil water storage explained the effect of different AI and CI IMs on ETa under the three IVs (**Figure 5**). Each harvest soil moisture value in 2017 was higher than the initial value under the AI treatment with 30 mm irrigation volume (**Figure 4**). The AI treatment soil water storage was 21 mm higher than the CI treatment soil water storage at the 3rd harvest with a 20 mm irrigation volume (**Figure 4**). Wang et al. (2021) also found that AI reduced the one-year-old alfalfa ETa, mainly caused by the alfalfa ETa at the 2^nd^ harvest. Although we did not measure soil evaporation, it was speculated that the low alfalfa coverage at the seedling stage increased soil evaporation and greatly reduced the soil water storage. Furthermore, our soil moisture monitoring revealed that deep soil water was being depleted, further confirming that no deep seepage had occurred, even though under 30 mm for AI (**Figure 4**). In most cases, reduced soil evaporation by decreasing the wetting degree of the rewatered soil (Du et al., 2017; Gong et al., 2005; Kang et al., 2003) or the implementation of AI could cut down ETa by making some leaves close their stomata or by decreasing the leaves’ stomatal opening degree to reduce plant transpiration (Abboud et al., 2019; Liu et al., 2021a; Liu et al., 2021b; Wang et al., 2016; Zhang et al., 2019). These are possibly the reasons why AI is widely used in many fruit trees and crops to reduce ETa (Abboud et al., 2019; Du et al., 2017; Gong et al., 2005; Kang et al., 2003; Liu et al., 2021; Liu et al., 2021a).

Interestingly, compared to CI, the alfalfa ETa of AI increased by 6–29 mm under a 10 mm irrigation volume in 2018 (**Figure 3**). Wang et al. (2021) also found that AI increased the two-year-old alfalfa ETa, mainly caused by the alfalfa ETa at the 3^rd^ harvest. The reduction in soil water storage among AI treatments was between 25 and 93 mm in 2018 (**Figure 5**), which may explain the increase in ETa in our experiment. AI increased the irrigation time interval, which causes the roots to absorb compensatory water after the rewetting event (Jovanovic et al., 2010; Liu et al., 2022; Tang et al., 2005) and may explain the increase in alfalfa ETa. AI is undeniably the application of an alternative irrigation method, but CI may also play a more advantageous role in other aspects. CI forms patches of soil water heterogeneity (Appels and Karimi, 2021; Kandelous et al., 2012) compared with AI in our experiment, suggesting that it may decrease the alfalfa ETa. Soil water heterogeneity can be formed due to the large spacing between two CI pipes when installing a CI system or the incomplete wetting of the whole soil layer by less irrigation volume (Kandelous et al., 2012, Kandelous et al., 2011). Although no CI-based AI study on field soil evaporation was found, a potted experiment using plastic syringes showed that soil evaporation under fixed AI (similar to CI in this study) was lower than that under AI, which is also a reason for the decrease in ETa under CI (Puértolas and Dodd, 2022). In addition, a shorter irrigation event time interval under CI reduced the dead roots compared to AI, reducing the investment in secondary roots after a rewetting event occurred (Li et al., 2011; Xiao et al., 2015). The reduction of secondary roots decreased the available water absorption in the wet soil part (Segovia-Cardozo et al., 2022) and may be inferred that CI decreased the alfalfa ETa. Consequently, these two IMs have their own advantages and disadvantages for alfalfa ETa; more extensive research is needed among different genotypes and plants.

The results also showed that the ETa of one-year-old alfalfa was 378–560 mm, while that of two-year-old alfalfa was 476–877 mm (**Figure 3**), wherein the ETa ranged from 421 to 877 mm in AI and from 378 to 835 mm in CI. The ETa of alfalfa in the year of establishment was significantly lower than that of biennial alfalfa. The biggest factor affecting this was the number of harvests, namely, only three harvests in 2017, while four harvests were carried out in 2018 (**Figure 1**). Therefore, the growing season for two-year-old alfalfa is longer than that for one-year alfalfa. This was close to the previous subsurface drip irrigation results of 498 mm at the same site (Kou, 2014). It is close to another flood irrigation range of 482–843 mm in the Hexi Corridor (Liu et al., 2022). These were much lower than the ETa of alfalfa measured by a lysimeter in other countries (Benli et al., 2006; Wright, 1988). Three explanations may justify the differences between our results and those of other research. The first reason is that our maximum irrigation volume may not reach the maximum evapotranspiration required by alfalfa. The second reason may be that the rainfall in our research area is scarce, which did not exceed 200 mm (109 mm in 2017 and 192 mm in 2018), while the rainfall in other studies was much greater than that at our research site. The third reason might be that alfalfa was harvested only 3–4 times in the Hexi Corridor (Wang et al., 2021; Liu et al., 2022; Wang et al., 2021), while there were more harvest events in other studies than in this study (Benli et al., 2006; Wright, 1988). In summary, reasonable irrigation and harvest management practices are important factors in understanding changes in alfalfa ETa. By carefully controlling these factors, it is possible to create consistent growing conditions that can facilitate accurate measurement and interpretation of changes in ETa over time.

ETa is also significantly affected by irrigation volume. In most cases, ETa is lower when irrigation volume is lower (**Figure 3**). Under subsurface drip irrigation conditions, lower irrigation volumes reduce the volume of topsoil moistened, which may decrease soil evaporation (Kandelous et al., 2012). The reduced soil moisture content may also limit water absorption by plants, leading to lower transpiration (Kandelous et al., 2012). Furthermore, biennial alfalfa has a deeper and denser root system than annual alfalfa (Ma et al., 2025). Both the deeper and denser roots may increase the root system’s ability to absorb water, thus improving the alfalfa’s ETa. Moreover, biennial alfalfa requires a higher total irrigation amount than annual alfalfa, which is another reason for its higher ETa (**Figure 5**).

### 4.2 AI increased alfalfa hay yield compared with CI

We found that AI improved the alfalfa hay yield under the 10 and 20 mm IVs compared with CI in two years (**Figure 3**). This result is consistent with that obtained by Gençoğlan et al. (2006), who showed that the green bean yield of AI increased by about 37% under the same irrigation volume as CI. Generally, AI is an alternate irrigation method that can maintain potato yield (Jovanovic et al., 2010). However, Çolak et al. (2018) demonstrated that AI significantly reduced eggplant yield compared with CI. These three comparative studies did not reach a consistent conclusion. We speculated that this was because the harvesting organs of the three crops differed. Seeds are harvested in green bean, tubers are harvested in potato, and a fruit-type organ is harvested in eggplant. In the alfalfa we studied, the shoots were harvested. The different organs that are harvested have different responses to AI and CI, which may also lead to inconsistent results. Sun et al (2024) research found that irrigation methods significantly increased alfalfa hay yield, which is consistent with our results (**Table 1**). Their study suggests that increased yield may be more significantly positively correlated with plant height (Sun et al., 2024). Similar results can be found in other study on alfalfa (Cao et al., 2025). Furthermore, alfalfa leaves may also contribute significantly to its yield, although we did not observe biomass allocation under AI. A partial root-zone drying trial based on furrow irrigation showed that under alternating furrow irrigation, more biomass was allocated to alfalfa leaves (Zhang et al., 2021). We speculate that this may be another reason why alfalfa yield is higher under AI than under CI. Our previous research has shown that AI significantly reduced one-year-old alfalfa yield without affecting two-year-old alfalfa yield (Wang et al., 2021). Wang et al. (2021) found that AI with full irrigation (30 mm/event) not only had limited effects on improving alfalfa hay yield but also reduced one-year-old alfalfa hay yield at the 3^rd^ harvest. We speculate that the advantages exhibited by AI are masked when fully irrigated. Subsequent findings revealed that AI’s advantages become apparent only under conditions of water deficit (Wang et al., 2024).

Soil water storage dynamics can partly explain the change in alfalfa hay yield with different irrigation methods. The soil water storage dynamics at a depth of 10–160 cm were significantly higher in AI than CI in two years (**Figure 4**). More water use in rhizosphere soil ensures the advantage of alfalfa yield under AI, which is different from the results of Çolak et al. (2018) and Wang et al. (2021). Çolak et al. (2018) placed subsurface drip pipes on both sides of eggplant under AI, and the distance between the two drip irrigation systems was 50 cm. In our study, the distance between the two subsurface drip irrigation systems under AI was 40 cm (**Figure 2**). Our subsurface drip irrigation arrangement comprised one drip irrigation pipe for four alfalfa rows, which differs from two drip irrigation pipes in one eggplant row. Thus, it can also explain the inconsistent results between their yields and ours under AI and CI. Another study at the same site found that AI significantly increased alfalfa soil water storage dynamics and shallow soil water content (10–100 cm), although it did not significantly increase alfalfa hay yield (Wang et al., 2021). Wang et al. (2021) showed that soil moisture did not have a good synchronicity in explaining yield variation under different irrigation regimes. Therefore, the impact of the two IMs on soil moisture and water storage dynamics, as well as their role in explaining alfalfa hay yield, still requires further study.

### 4.3 AI improved the alfalfa–water production function compared with CI

We also compared the relationship between hay yield and alfalfa ETa under the two irrigation methods, which is the key to measuring the alfalfa–water production function. Our results showed that compared with CI, AI could strengthen the slope of the relationship between alfalfa ETa and hay yield (**Figure 6**), which improved the alfalfa–water production function. Studies have shown that AI increases the slope of the green bean–water production function compared with CI (Gençoğlan et al., 2006), which is consistent with our results (**Figure 6**). A recent study has shown that AI decreases the slope of the eggplant–water production function compared with CI (Çolak et al., 2018). These inconsistent conclusions suggest that we should be cautious when selecting crops when applying CI to implement AI.

The results also revealed that the improvement in water production function due to AI stemmed more from increased yield than from actual crop evapotranspiration (**Supplementary Table 7**). We previously discussed that the advantage of ETa over CI under AI was not significant in section 4.1, but we subsequently found that AI showed a clear yield-increasing advantage in section 4.2. These further answers our hypothesis (ii) that AI improves the water production function by increasing alfalfa hay yield.

Although we did not find any existing studies to support AI that could strengthen the slope of the relationship between alfalfa ETa and hay yield, some studies have shown that there is a linear relationship between hay yield and alfalfa ETa under CI (Klocke et al., 2013; Saeed and El-Nadi, 1997). Other studies have shown a quadratic linear relationship of a single peak between hay yield and alfalfa ETa under CI (Lamm et al., 2012). These studies have shown that the alfalfa–water production function is complex under CI. It is necessary to conduct more studies on the alfalfa–water production function under different soil types, climatic conditions, and irrigation methods. Conducting further studies should help identify the optimal irrigation method and management practices for maximizing hay yield while minimizing water use. This could have important implications for sustainable agriculture, as efficient water use is critical for reducing the environmental impact of agriculture and ensuring long-term productivity.

### 4.4 Research limitations and prospects

The experimental comparison confounds irrigation alternation with drip-line configuration. CI used one drip system with wider spacing, whereas AI used two systems with narrower spacing. Therefore, the observed effects may reflect both irrigation alternation and differences in wetting geometry. In the future, more refined field experiments with separated root systems should be conducted to more accurately explore the advantages and disadvantages of the two irrigation methods.

Although we determined that the difference in ETa between the two irrigation methods might be influenced by irrigation volume and plant age, the limitations of our research at the time prevented us from effectively distinguishing between root uptake, soil evaporation, crop transpiration, and deep percolation. The importance of these three components in explaining plant ETa is self-evident, although some studies have attempted to effectively differentiate their contributions to explaining ETa using pot experiments (Puértola and Dodd, 2022). However, more field trials are needed to demonstrate the contribution of root uptake, soil evaporation, crop transpiration, and deep percolation to explaining ETa of AI in pot experiments (perhaps stable isotopes would be a better approach).

## 5 Conclusion

The comparison between the CI and AI methods in two years showed that the two IMs had their own advantages and disadvantages in alfalfa ETa, and AI had advantages in enhancing hay yield under low IVs (10 and 20 mm) and the alfalfa–water production function. AI decreased one-year-old alfalfa ETa under 20 and 30 mm irrigation volumes, which can be explained by soil moisture and soil water storage dynamics. However, CI reduced the alfalfa ETa under a 10 mm irrigation volume in 2017 and under a 30 mm irrigation volume in 2018. AI also strengthened the slope of the alfalfa–water production function. The increase is largely explained by the improved hay yield under AI treatment. These findings suggest that AI is a better irrigation method to improve alfalfa hay yield and the alfalfa–water production function in most cases, but the advantages and disadvantages of ETa under AI and CI should be weighed in the process of practical application. We may focus on comparing the economic benefits of the two IMs in future explorations. In the final analysis, AI used more drip irrigation pipes.

## Data Availability

The original contributions presented in the study are included in the article/**Supplementary Material**. Further inquiries can be directed to the corresponding authors.
