# Supplementary material for: Alternate partial root zone subsurface drip irrigation increases alfalfa water production function by increasing hay yield, rather than actual crop evapotranspiration
Source: Front Plant Sci. 2026 Aug 26;17:1909849. doi: 10.3389/fpls.2026.1909849 (PMC13553455; doi:10.3389/fpls.2026.1909849)
Supplement: Supplementary file 1 [file SupplementaryFile1.docx]

|  | **Supplementary Table 1. Type III fixed effects test of ETa** | | | | | | | | | | | |  |
| --- | --- | --- | --- | --- | --- | --- | --- | --- | --- | --- | --- | --- | --- |
|  | Source | Numerical degrees of freedom | | | Denominator degrees of freedom | | | | F | | Significance | |  |
|  | Intercept | 1 | | | 104.397 | | | | 10.492 | | .002 | |  |
|  | Irrigation method | 1 | | | 411.702 | | | | .048 | | .827 | |  |
|  | Irrigation volume | 2 | | | 119.000 | | | | 23.273 | | <.001 | |  |
|  | Stand year | 1 | | | 104.399 | | | | 10.459 | | .002 | |  |
|  | Harvest time | 1 | | | 97.265☑ | | | | 19.140 | | <.001 | |  |
|  |  | | | | | | | | | | | |  |
|  | **Supplementary Table 2. Fixed effects estimation of ETa** | | | | | | | | | | | |  |
|  | Parameter | | Estimation | Standard error | | Degrees of freedom | t | Significance | | 95% confidence interval | | | |
|  |  |  |  |  |  |  |  |  |  | Lower limit | | Upper limit | |
|  | Intercept | | 142983.554 | 44130.549 | | 104.397 | 3.240 | .002 | | 55474.936 | | 230492.171 | |
|  | [Irrigation method=AI] | | -2.579 | 11.765 | | 411.702 | -.219 | .827 | | -25.706 | | 20.548 | |
|  | [Irrigation method=CI] | | 0b | 0 | | . | . | . | | . | | . | |
|  | [Irrigation volume=10.00] | | -72.066 | 10.565 | | 119.000 | -6.821 | <.001 | | -92.986 | | -51.145 | |
|  | [Irrigation volume=20.00] | | -34.763 | 10.565 | | 119.000 | -3.290 | .001 | | -55.683 | | -13.843 | |
|  | [Irrigation volume=30.00] | | 0b | 0 | | . | . | . | | . | | . | |
|  | Stand year | | -70.738 | 21.873 | | 104.399 | -3.234 | .002 | | -114.111 | | -27.366 | |
|  | Harvest time | | -82160.653 | 18780.092 | | 97.265 | -4.375 | <.001 | | -119432.656 | | -44888.651 | |
|  | **Supplementary Table 3. Type III fixed effects test of Hay yield** \| \| \| \|; Source \| Numerical degrees of freedom \| Denominator degrees of freedom \| F \| Significance; Intercept \| 1 \| .000 \| 52.606 \| 1.000; Irrigation method \| 1 \| 31058666763.583 \| .000 \| .991; Irrigation volume \| 2 \| 43.258 \| 21.223 \| <.001; Stand year \| 1 \| .000 \| 36.321 \| 1.000; Harvest time \| 0 \| . \| . \| . | | | | | | | | | | | | |
| **Supplementary Table 4. Fixed effects estimation of hay yield** | | | | | | | | | | | | | |
| Parameter | | | Estimation | Standard error | | Degrees of freedom | t | Significance | | 95% confidence interval | | | |
|  |  |  |  |  |  |  |  |  |  | Lower limit | | Upper limit | |
| Intercept | | | 3355455.041 | 462776.191 | | .000 | 7.251 | 1.000 | | -3844358.246 | | 10555268.328 | |
| [Irrigation method=AI] | | | -352.463 | 32672.664 | | 31058666763.583 | -.011 | .991 | | -64389.709 | | 63684.782 | |
| [Irrigation method=CI] | | | 0 | 0 | | . | . | . | | . | | . | |
| [Irrigation volume=10.00] | | | -1579.800 | 244.456 | | 43.258 | -6.463 | <.001 | | -2072.707 | | -1086.893 | |
| [Irrigation volume=20.00] | | | -964.710 | 244.456 | | 43.258 | -3.946 | <.001 | | -1457.617 | | -471.802 | |
| [Irrigation volume=30.00] | | | 0 | 0 | | . | . | . | | . | | . | |
| Stand year | | | -1660.716 | 275.561 | | .000 | -6.027 | 1.000 | | -4915.181 | | 1593.749 | |
| Harvest time | | | 8.980E-13 | 1.490E-13 | | .000 | 6.027 | 1.000 | | -8.618E-13 | | 2.658E-12 | |
|  | | | | | | | | | | | | | |
|  |  | | | | | | | | | | | | |

| **Supplementary Table 5. Multivariate test^a^** | | | | | | | |
| --- | --- | --- | --- | --- | --- | --- | --- |
| Effect | | Value | F | Hypothesis degrees of freedom | Error degrees of freedom | Significance | Partial Eta Squared |
| Intercept | Biley's trajectory | .000 | .^b^ | .000 | .000 | . | . |
|  | Wilker Lambda | 1.000 | .^b^ | .000 | 117.500 | . | . |
|  | Hotlin's trajectory | .000 | .^b^ | .000 | 2.000 | . | . |
|  | Roy's maximum root | .000 | .000^b^ | 2.000 | 116.000 | 1.000 | .000 |
| Irrigation method | Biley's trajectory | .103 | 6.697^b^ | 2.000 | 117.000 | .002 | .103 |
|  | Wilker Lambda | .897 | 6.697^b^ | 2.000 | 117.000 | .002 | .103 |
|  | Hotlin's trajectory | .114 | 6.697^b^ | 2.000 | 117.000 | .002 | .103 |
|  | Roy's maximum root | .114 | 6.697^b^ | 2.000 | 117.000 | .002 | .103 |
| Irrigation volume | Biley's trajectory | .637 | 27.572 | 4.000 | 236.000 | <.001 | .318 |
|  | Wilker Lambda | .367 | 38.031^b^ | 4.000 | 234.000 | <.001 | .394 |
|  | Hotlin's trajectory | 1.711 | 49.628 | 4.000 | 232.000 | <.001 | .461 |
|  | Roy's maximum root | 1.705 | 100.568^c^ | 2.000 | 118.000 | <.001 | .630 |
| Stand year | Biley's trajectory | .000 | .^b^ | .000 | .000 | . | . |
|  | Wilker Lambda | 1.000 | .^b^ | .000 | 117.500 | . | . |
|  | Hotlin's trajectory | .000 | .^b^ | .000 | 2.000 | . | . |
|  | Roy's maximum root | .000 | .000^b^ | 2.000 | 116.000 | 1.000 | .000 |
| Harvest time | Biley's trajectory | .000 | .^b^ | .000 | .000 | . | . |
|  | Wilker Lambda | 1.000 | .^b^ | .000 | 117.500 | . | . |
|  | Hotlin's trajectory | .000 | .^b^ | .000 | 2.000 | . | . |
|  | Roy's maximum root | .000 | .000^b^ | 2.000 | 116.000 | 1.000 | .000 |
| Irrigation method * Irrigation volume | Biley's trajectory | .020 | .599 | 4.000 | 236.000 | .664 | .010 |
|  | Wilker Lambda | .980 | .596^b^ | 4.000 | 234.000 | .666 | .010 |
|  | Hotlin's trajectory | .020 | .594 | 4.000 | 232.000 | .668 | .010 |
|  | Roy's maximum root | .019 | 1.149^c^ | 2.000 | 118.000 | .320 | .019 |
| Stand year * Harvest time | Biley's trajectory | .000 | .^b^ | .000 | .000 | . | . |
|  | Wilker Lambda | 1.000 | .^b^ | .000 | 117.500 | . | . |
|  | Hotlin's trajectory | .000 | .^b^ | .000 | 2.000 | . | . |
|  | Roy's maximum root | .000 | .000^b^ | 2.000 | 116.000 | 1.000 | .000 |
| ^a^. Design: Intercept + Irrigation method + Irrigation volume + Stand year + Harvest time + Irrigation method * Irrigation volume + Stand year * Harvest time | | | | | | | |
| ^b^. Precise statistics | | | | | | | |

^c^. This statistic is the upper limit of F that generates the lower limit of significance level.

| **Supplementary Table 6. Intersubjective effects test** | | | | | | | |
| --- | --- | --- | --- | --- | --- | --- | --- |
| Source | Dependent variable | Type III sum of squares | Degrees of freedom | Mean square | F | Significance | Partial Eta Squared |
| Corrected model | ETa | 189609.420^a^ | 7 | 27087.060 | 9.871 | <.001 | .369 |
|  | Hay yield | 109839728.785^b^ | 7 | 15691389.826 | 53.863 | <.001 | .762 |
| Intercept | ETa | .000 | 0 | . | . | . | .000 |
|  | Hay yield | .000 | 0 | . | . | . | .000 |
| Irrigation method | ETa | 209.476 | 1 | 209.476 | .076 | .783 | .001 |
|  | Hay yield | 3913261.167 | 1 | 3913261.167 | 13.433 | <.001 | .102 |
| Irrigation volume | ETa | 109108.233 | 2 | 54554.116 | 19.881 | <.001 | .252 |
|  | Hay yield | 53266763.189 | 2 | 26633381.595 | 91.423 | <.001 | .608 |
| Stand year | ETa | .000 | 0 | . | . | . | .000 |
|  | Hay yield | .000 | 0 | . | . | . | .000 |
| Harvest time | ETa | .000 | 0 | . | . | . | .000 |
|  | Hay yield | .000 | 0 | . | . | . | .000 |
| Irrigation method * Irrigation volume | ETa | 2335.964 | 2 | 1167.982 | .426 | .654 | .007 |
|  | Hay yield | 539676.176 | 2 | 269838.088 | .926 | .399 | .015 |
| Stand year * Harvest time | ETa | .000 | 0 | . | . | . | .000 |
|  | Hay yield | .000 | 0 | . | . | . | .000 |
| Error | ETa | 323798.170 | 118 | 2744.052 |  |  |  |
|  | Hay yield | 34375672.380 | 118 | 291319.257 |  |  |  |
| Total | ETa | 3828113.650 | 126 |  |  |  |  |
|  | Hay yield | 1221972615.840 | 126 |  |  |  |  |
| Revised total | ETa | 513407.590 | 125 |  |  |  |  |
|  | Hay yield | 144215401.165 | 125 |  |  |  |  |
| a. R^2^ = .369（Adjusted R^2^ = .332） | | | | | | | |
| b. R^2^ = .762（Adjusted R^2^ = .747） | | | | | | | |

| **Supplementary Table 7.** **Paired comparison** | | | | | | | |
| --- | --- | --- | --- | --- | --- | --- | --- |
| Dependent variable | (I) Irrigation method | (J) Irrigation method | Mean difference (I-J) | Standard error | Significance^c^ | 95% confidence interval^c^ | |
|  |  |  |  |  |  | Lower limit | Upper limit |
| ETa | AI | CI | -2.579^a^ | 9.333 | .783 | -21.061 | 15.904 |
|  | CI | AI | 2.579^a^ | 9.333 | .783 | -15.904 | 21.061 |
| Hay yield | AI | CI | -352.463^a,*^ | 96.168 | <.001 | -542.902 | -162.025 |
|  | CI | AI | 352.463^a,*^ | 96.168 | <.001 | 162.025 | 542.902 |
| Based on the estimated marginal means | | | | | | | |
| ^*^. The significance level of the difference in means is .05. | | | | | | | |
| ^a^. Although the adjusted group marginal means (I) and (J) are not estimable, the difference (I-J) is estimable.  ^c^. Multiple comparison adjustment: minimum significance difference method (equivalent to no adjustment). | | | | | | | |
